# Implementation challenges and successes of a population-based colorectal cancer screening program: a qualitative study of stakeholder perspectives

**DOI:** 10.1186/s13012-015-0227-z

**Published:** 2015-03-29

**Authors:** Elizabeth G Liles, Jennifer L Schneider, Adrianne C Feldstein, David M Mosen, Nancy Perrin, Ana Gabriela Rosales, David H Smith

**Affiliations:** Center for Health Research, Kaiser Permanente Northwest, 3800 N. Interstate Ave, Portland, OR 97227 USA; Northwest Permanente, Kaiser Permanente Northwest, 500 NE Multnomah St, Suite 100, Portland, OR 97232 USA

**Keywords:** Colorectal cancer screening, Fecal immunochemical testing, PRISM, Screening facilitators, Screening barriers, Centralized screening program

## Abstract

**Background:**

Few studies describe system-level challenges or facilitators to implementing population-based colorectal cancer (CRC) screening outreach programs. Our qualitative study explored viewpoints of multilevel stakeholders before, during, and after implementation of a centralized outreach program. Program implementation was part of a broader quality-improvement initiative.

**Methods:**

During 2008–2010, we conducted semi-structured, open-ended individual interviews and focus groups at Kaiser Permanente Northwest (KPNW), a not-for-profit group model health maintenance organization using the practical robust implementation and sustainability model to explore external and internal barriers to CRC screening. We interviewed 55 stakeholders: 8 health plan leaders, 20 primary care providers, 4 program managers, and 23 endoscopy specialists (15 gastroenterologists, 8 general surgeons), and analyzed interview transcripts to identify common as well as divergent opinions expressed by stakeholders.

**Results:**

The majority of stakeholders at various levels consistently reported that an automated telephone-reminder system to contact patients and coordinate mailing fecal tests alleviated organizational constraints on staff’s time and resources. Changing to a single-sample fecal immunochemical test (FIT) lessened patient and provider concerns about feasibility and accuracy of fecal testing. The centralized telephonic outreach program did, however, result in some screening duplication and overuse. Higher rates of FIT completion and a higher proportion of positive results with FIT required more colonoscopies.

**Conclusions:**

Addressing barriers at multiple levels of a health system by changing the delivery system design to add a centralized outreach program, switching to a more accurate and easier-to-use fecal test, and providing educational and electronic support had both benefits and problematic consequences. Other health care organizations can use our results to understand the complexities of implementing centralized screening programs.

## Background

Colorectal cancer (CRC) is the third most common non-skin cancer, and the second leading cause of cancer-related death, in the U.S. [1]. The early detection of high-risk lesions or of CRC itself through appropriate screening is associated with decreased incidence of and mortality from CRC [2-4]. Currently, recommended CRC screening modalities for average-risk patients include annual fecal occult blood testing (FOBT) or colonoscopy every 10 years, or flexible sigmoidoscopy every 5 years, with or without interval FOBT [5]. In addition, as a result of improved test performance and usability, in 2008, multiple professional societies endorsed the use of four types of fecal immunochemical tests (FIT) to replace guaiac FOBT for CRC screening [6].

Despite the proven benefits, CRC screening at regular intervals for age-appropriate populations remains sub-optimal in the U.S.. Although more than half of adults aged 50 and older in the U.S. have ever received a CRC screening test, millions of Americans are not receiving CRC screening at all, or are not repeating screening tests at recommended intervals [7]. Population-based screening programs that employ patient-screening reminders [8-11], provision of stool tests to complete at home [12], and provider feedback are effective for improving uptake of CRC screening [13]. Prior studies in systems in the U.S. that offer more than one type of screening test (i.e., both endoscopy and fecal testing) demonstrate that one-to-one counseling of patients by nurses, patient navigators, or health educators also modestly increase screening rates [4,14,15]. However, there are few studies [15] on system-level challenges, or on determinants of success, encountered in the process of implementing such programs.

We conducted a qualitative case study [16-18] to explore the viewpoints of multilevel stakeholders implementing a centralized population-based CRC screening program and quality-improvement initiative in an integrated health system in the U.S. between 2009 and 2011. At the study site, screening flexible sigmoidoscopy and guaiac-based FOBT were available as screening options until 2008, when the health system began offering an additional option of screening colonoscopy. Within the system, the primary care provider provides an electronic fecal test order (a process termed “in-reach”) or a referral to specialty care for flexible sigmoidoscopy or colonoscopy when seeing the patient in a clinic visit. Our previously published randomized trial [19] (ClinicalTrials.gov identifier: NCT00656838) comparing an automated telephone contact campaign encouraging fecal test screening to clinic-based encouragement of fecal testing (usual care) found that those in the intervention group were significantly more likely to complete a fecal test (hazard ratio, 1.31; 95% confidence interval, 1.10–1.56) compared with usual care. In 2008, the health system implemented this automated telephone contact campaign and in 2009, enabling a centralized order for a fecal test (a process termed “outreach”) and direct mailing of the kits to interested patients. The health system also implemented several related quality initiatives supporting colorectal cancer screening and switched from guaiac fecal occult blood testing to fecal immunochemical testing (FIT) around the same time. Our study used a combination of semi-structured, open-ended individual interviews and focus groups. We aimed to provide sufficient in-depth description from stakeholder groups to generate breadth and depth of information, and to maximize trustworthiness and credibility of data [20,21]. We also investigated specific barriers and facilitators of colorectal cancer screening from stakeholder perspectives at different levels within the organization, and explored unintended consequences of the program.

## Methods

### Study site and data systems

We conducted our qualitative study between 2008 and 2010 at Kaiser Permanente Northwest (KPNW), a not-for-profit group model health maintenance organization (HMO) with about 485,000 members in Southern Washington and the Portland, Oregon, metro area. KPNW regional electronic databases capture >95% of all medical care and pharmacy services members receive, and data are linked through each member’s health record number. A full electronic medical record (EMR) has been in place since 1996. The study design and procedures were approved by KPNW’s institutional review board, and interview participants provided written informed consent.

### CRC phone-reminder program and surrounding quality-improvement activities

Our qualitative study was designed to take advantage of an already in-process change in care practices at KPNW. In 2008, the organization implemented a new CRC screening-reminder program and related quality-improvement initiatives. The reminder program used automated phone calls to encourage CRC screening for average-risk, age-appropriate members who were coming due, all identified through diagnosis and procedural codes in the electronic medical record. Details on the design and outcomes of this program are reported elsewhere [19]. Briefly, the automated reminder call was about 1 min in length and provided an overview of the benefits of CRC screening. Recipients could request fecal test kits by pressing a number via touch-tone telephone. The automated system had an algorithm for accepting calls back, leaving messages, and for reminding those who had already received a kit in the mail to complete it.

Other quality-enhancement efforts within the organization included heightened training of providers (through in-person education, decision support tools in the EMR, and evidence-based practice guidelines) highlighting CRC screening importance and methods. The organization also implemented staff financial incentives for meeting organizational target goals in CRC screening rates.

### Conceptual framework for evaluating local practices in a larger context

**Figure 1:**
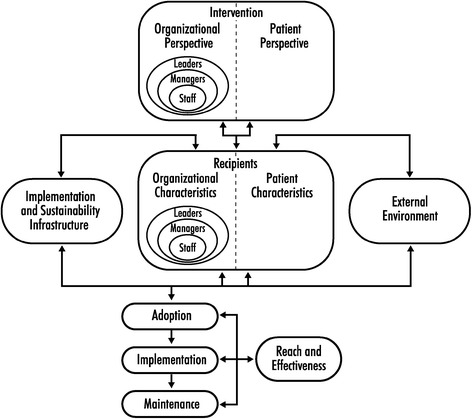
**The practical robust implementation and sustainability model (PRISM).** The PRISM model for integrating research findings into practice considers how the program or intervention design, the external environment, the implementation and sustainability infrastructure, and the recipients (especially at the level of health care providers and their support staff) influence program adoption, implementation, and maintenance.

**Figure 2:**
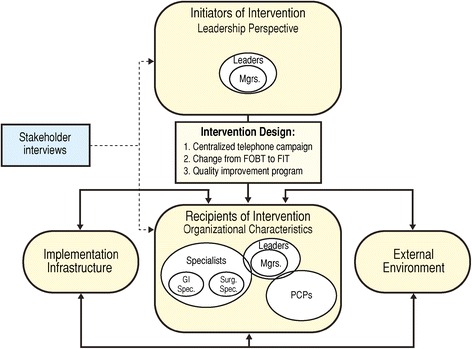
**A qualitative study of stakeholder assessment of a colorectal cancer screening program by PRISM domains.** This adapted PRISM diagram outlines how stakeholder interviews among both the initiators and recipients of a multimodal program of colorectal cancer screening evaluated the screening program. It shows the relative roles of the stakeholder groups as initiators (leadership and management) and/or recipients (specialists, primary care providers, leaders, and managers) of the intervention. Stakeholders reflected upon the historical barriers and facilitators to colorectal cancer screening, considering characteristics of the organization itself, external environment and implementation infrastructure of the organization. They also gave input into the successes of the intervention and described remaining challenges, in relation to the organizational characteristics.

The guiding framework for our study was the practical robust implementation and sustainability model (PRISM [22], presented in Figure 1). PRISM advocates for documenting and defining key factors or “leverage points” at multiple levels of internal and external stakeholder influence. The model considers how the external environment, intervention design, implementation infrastructure, and adopting organization (with particular emphasis on the health care teams and providers) and its patients influence program implementation and success. PRISM expands upon the RE-AIM framework [23,24], derived from work in the diffusion of innovations [25-27], and is supported by social ecology and the PRECEDE/PROCEED model [23,28]. The adaptation of PRISM for this study is illustrated in Figure 2.

### Study participants and recruitment

We gathered qualitative data from 55 participants from different levels of the organization: 8 health plan leaders, 4 program managers, 23 endoscopy specialists (15 gastroenterologists, 8 general surgeons), and 20 primary care providers (PCPs).

We identified health plan leaders and managers based on their role in the organization as a resource and program decision-maker, and/or their role in designing and implementing the CRC screening program. We invited all health plan leaders through an organizational e-mail to participate in a 45-min face-to-face interview, and provided lunch for participants. All of them agreed to participate. We interviewed four of them prior to the intervention and four of them after the intervention, to obtain perspectives over time on the anticipated and realized successes and challenges of the implementation. We also invited four managers designing and implementing the centralized CRC screening program (*n* = 4) to a 90-min focus group, occurring after the intervention; all of them participated.

We identified gastroenterologists and general surgeons because they conduct CRC screening endoscopy (colonoscopy and flexible sigmoidoscopy) within the organization. We obtained permission to conduct a 30-min focus group with the gastroenterologists during a regional staff meeting; attendance at the meeting was mandatory but participation in the focus group was optional (with informed consent obtained). All 15 gastroenterologists present at the meeting participated. We conducted a similar but separate focus group for the general surgeons; 8 out of 11 general surgeons present actively participated, while 3 listened but did not share in the discussion. These focus groups occurred toward the end of the intervention phase.

We identified and interviewed 20 PCPs [20,29,30] who had different practice emphases on CRC screening test type and varying overall screening rates. We generated a list of family practice (FP) and internal medicine (IM) providers who had adult patient panels in 2008 (n = 250) with at least 20 patients eligible for CRC screening (*n* = 246). We identified “lower” (<20th percentile) and “higher” (>80th percentile) performers based upon their 2008 CRC screening-performance rates ascertained through routine HEDIS quality measures by the health plan. We further divided the list into those who used more stool tests and those who used more endoscopy, based on proportion of completed tests. From this process, we identified a total of 102 PCPs and sent out 67 individual recruitment emails to a representative subgroup of the identified list to reach a total of 20; both PCPs and the staff recruiting and conducting the interviews were blinded to whether they fell into the “lower” or “higher” screening performance groups. Forty-one participants provided no response to the email invitation, and 6 participants indicated scheduling conflicts or lack of time as their reason for not participating.

### Data collection and analysis

A trained, third-party qualitative methodologist not known to the participants [JS] conducted interviews, and interviews were audio-recorded and professionally transcribed for analysis. Focus groups were facilitated by both EL and JS, with notes taken manually by JS. Interview and focus group questions elicited information about stakeholders’ perspectives regarding barriers and facilitators to CRC screening in general, and specifically within the organization; overall reaction to the organization’s CRC screening program, including to the use of automatic reminder calls and stool tests. Questions also elicited advice on improving the CRC screening program. The research team developed and refined interview and focus group guides using an iterative approach, whereby team members regularly met to discuss themes generated by prior interviews and to consider areas of exploration for subsequent interviews [30-32]. Coauthor JS led the analyses, with guidance and input from the research team. We used a content analysis approach, aided by the use of a qualitative-research software package, ATLAS.ti 5.0 (Scientific Software Development, 1997) to code data and generate reports of coded text for analysis. We developed a coding dictionary based on the interview guide and review of the transcribed interviews. Transcribed interviews were coded by marking passages of text with phrases indicating content of the discussions. Using the report and query functions of ATLAS.ti, we reviewed coded text through a deductive process, resulting in refined themes [29,33,34].

## Results and discussion

### Historical barriers to CRC screening

#### Unclear screening test emphasis

The most commonly perceived external (non-organizational) barrier expressed was that expert opinions about the best screening option for low- or average-risk patients were inconsistent. Internal CRC screening recommendations had changed over the course of 5 years, with an emphasis on sigmoidoscopy initially, followed by an emphasis on stool testing, followed by allowance of multiple options. Health plan leaders/managers stated that there were too many screening options in the system (i.e., flexible sigmoidoscopy, colonoscopy, and fecal testing), with limited guidance for providers and patients about which test to choose. Specialists and health plan leaders/managers alike perceived that PCPs, influenced by training or work culture, may promote primarily screening colonoscopies rather than stool tests or sigmoidoscopy as options for average/low-risk patients. Similarly, PCPs and some of the specialists expressed discomfort with the previous years’ focus on stool tests and flexible sigmoidoscopy as preferred screening options, rather than colonoscopy, because this emphasis did not match community norms and expectations. Primary care providers expressed that they received a “mixed-message” from the organization by being allowed to refer patients to colonoscopy to screen for CRC in average-risk patients, but then were not always supported by health plan leaders or specialists for doing so. They also said that frequent changes in the emphasis on screening options for patients created confusing expectations (Table 1).

**Table 1:** **Historical barriers to CRC screening (** ***n*** **= 55)**

**Summary of individual themes related to organizational challenges**	**Sample of illustrative quotes (stakeholder group identified)**
Unclear evidence on choosing screening tests	
Too many options in the system for screening and no clear guidelines for providers or patients	“It’s amazing the paucity of evidence around what’s really the best test. The stool cards have been tested more rigorously than other interventions, so we know more about that. But that doesn’t necessarily mean we know that colonoscopy is not as good.” —HP leader
Mixed-message received from health plan because of allowing referral for screening colonoscopies, but not having full support to get the colonoscopies done	“Initially, there was tremendous resistance to doing colonoscopies on people that didn’t have a first degree relative with a history of colon cancer. And, we were under-utilizing the hemoccults. But we would get into a twenty minute debate with a patient who wanted a colonoscopy… So, I never know what’s right or whether our system just had it’s resources in the wrong place. First they tell you to do one thing in the system, then it changes… it makes you dizzy.” —PCP
Prior organizational focus on fecal tests and flexible sigmoidoscopy not matching community standard or national recommendations	“The community standard for screening is colonoscopy as recommended by the American Society of Gastroenterologists… Then [patients] say, ‘Well, the Internet’s kind of said that that’s really the best thing to do.’ And then we have to say, ‘Well, we’re not offering that to you.’ And that can be quite contradictory. And having that conversation can be quite challenging.” *—*General surgeon
PCPs and specialists influenced by training or culture promote only screening colonoscopy and not other options (e.g., fecal test) for low-risk patients	“A lot of the younger primary care docs… were influenced by… one of the leaders in the field… [The] one lecture a year he gave to the house staff was that colonoscopy is the way to go.” *—*GI specialist
Colonoscopy resource constraints	
Restricted access to screening colonoscopy within the organization	“How tight the access issue is, is an ongoing sort of challenge and frustration for the GI department.” *—*GI specialist
PCPs ordering screening colonoscopies when the patient is symptomatic, rather than as a diagnostic test, complicates triaging a limited resource	“I think our system would benefit if we actually went back to basics… It seems like we get a lot of referrals for screening when the patient has abdominal bloating or they have diarrhea. It’s not clear if the other person on the other end understands what the term screening really means… That really blurs the triaging to try to figure out which patients to see first and get things done effectively.” *—*GI specialist
Over-screening the already screened or offering screening to those who may not need it (e.g., patient on hospice care) complicates triaging a limited resource	“People with a life expectancy of less than five years, it makes absolutely no sense to offer them colon cancer screening, but we see this all the time. Or if they have Class 4 heart failure, or if they have some other cancer that has failed chemotherapy and they’re on hospice.” *—*GI specialist
Primary care and specialty department constraints	
Lack of time during office visit and addressing patients’ competing demands makes thoroughly discussing CRC screening and options difficult	“I find it hard when someone is in for something else and these [CRC screening] orders get pended, that I don’t feel like talking about in that visit because they’ve just been diagnosed with diabetes or there’s something really pressing going on that I need to talk about with the patient… it’s not the time to talk about colon cancer screening.” —PCP
Hard to negotiate both patient demand and offer “choice” of test while also honoring organizational emphasis on fecal testing	“As a clinician here, since we aren’t pushing or embracing the idea of colonoscopy as primary screening, the conversations I end up having to have with patients who want colonoscopy [involve] talking about a long wait time in getting them a colonoscopy if they want it, even though it’s not our first recommendation.” —PCP
Referral process for a screening colonoscopy involves multiple steps and departments, which sometimes creates miscommunication and lack of follow-up	“The referral is more challenging than for something like a Pap, which I can do it when they come in. I have more control over that. As opposed to CRC screening [colonoscopy] and having to send in a referral, having the patient be called back or a letter sent. It’s just more steps to get in.” —PCP
Specialists tend to have a limited historical role in helping to shape organization’s CRC screening approach	“The (surgery) department hasn’t really provided any leadership around influencing colon cancer screening. They’ve played a passive role, for the most part, in supporting what was the flex sig [sic] program as an orphan department. I don’t recollect surgeons being on the colon cancer screening meetings for the last number of years.” —General surgeon

#### Colonoscopy resource constraints

Stakeholders also uniformly expressed concern about limited availability of trained specialists for conducting screening colonoscopies. Surgery and gastroenterology specialists said that inappropriate referrals for colonoscopy further strained already limited colonoscopy resources. Primary care providers reported that resource constraints created challenges with patient rapport, due to having to explain and negotiate the long wait for access to a screening colonoscopy. They described as challenging working in an environment that had undergone numerous shifts in how the organization allocated CRC screening resources.

#### Primary care and specialty department constraints

PCPs felt specialists lacked appreciation or awareness of the time constraints and pressures they are under during a 20-min office visit, making it challenging to always thoroughly discuss with patients CRC screening importance, risk/benefits, and various screening options. The challenge of offering patients a choice in the discussion of CRC screening options compounded the problem. PCPs felt a lack of support in ensuring that patients followed through with screening completion. They felt the communication from support staff to patients about how to return completed stool test kits was inconsistent and confusing. Additionally, PCPs felt the referral process for a screening colonoscopy involved too many steps and departments, leaving room for miscommunication, misunderstanding, and lack of follow-up with the patient. Specialists acknowledged that they had historically tended to have a limited role in shaping the organizations CRC screening program.

### Historical facilitators to CRC screening

#### Historical emphasis on prevention

Health plan leaders and managers described a historical focus on quality and prevention in the organization’s mission as a facilitator of continued efforts to improve CRC screening rates. They were spurred on by the fact that the local organization’s quality performance numbers for CRC screening had historically been lower than those in other regions, who had demonstrated improvement by adopting a more formalized and centralized outreach program. They also reported prior successes in improving screening rates for other prevention measures (e.g., mammography screening) through a centralized reminder program as a strong motivator to continue to innovate. Health plan leaders cited this example as inspiration that a sustainable and cost-effective population-based CRC screening program acceptable to both providers and patients was possible (Table 2).

**Table 2:** **Historical facilitators to CRC screening (** ***n*** **= 55)**

**Summary of themes related to organizational facilitators**	**Sample of illustrative quotes (stakeholder group identified)**
Organization’s historical emphasis on prevention	
Overall focus on quality and prevention as a primary part of organization’s mission and values	“The one thing we don’t argue is that we need screening of some type for colon cancer. Everyone knows the old adage is that any screen is better than no screening. So we all agree that we need to get there to screen the population. And we’ve got to decide what’s the best way to do it for our population.” —GI specialist
Internal success at raising screening rates for other health issues (e.g., mammography for breast cancer screening) using a centralized outreach reminder approach	“Clearly, we had great results with breast cancer screening, and we had some good results with cervical cancer too… So that was part of what we wanted to test, does IVR calling work as well [for CRC]?” —HP leader
Quality performance numbers for CRC screening were not as good as other comparable health care organizations	“We saw what our screening rates were… and we looked around at other regions to see what they were doing successfully. Mid-Atlantic had used [interactive voice recognition]. And so I worked really closely with Mid-Atlantic to find out which IVR they used and what their success rate was with CRC screening… ” —HP leader
Preexisting integrated structure for dissemination of key practices	
Trust in the structure of the integrated health system to enable alignment of evidence-based CRC screening approaches with available resources and department roles	“And I know that, you know, we had a very strong analyst. We had a very strong negotiator. We had a strong physician lead who was very interested and extremely engaged. And then we had a project manager, I mean, that could just kind of manage all the pieces and make sure that everybody shows up and things are done in a timeline.” —HP leader
Strong trust in the skill level, training, and recommendations of endoscopy specialists	“I think that the GI doctors are just so dang ethical and skilled… they’re not going to recommend something just to save the organization money, and they’re still going to have the patient’s best interest in mind.” —PCP
Use of support staff (medical assistants) trained in educating and motivating patients on screening and follow-up	“We have our own MAs and own staff and we can say, okay, when a patient checks in and they’re due for one of these, you hand them this. If there’s no need, not involving the physician just speeds up things. If you have a nice handout and your staff is knowledgeable about the task and can explain it to somebody, like an MA, there’s no reason for taking time out of an appointment for the physician to go over the test, when the patient is there for something else. So finding the earliest person who is able to deliver the message early on is better.” —HP leader
Presence of PCP champions to assist other providers in navigating and integrating latest research with organizational goals and patient demand	“Presentations and talks [with clinician champion] have really been helpful. They have helped me kind of frame my conversations about everything… having a clinician who has looked at the research is really powerful.” —PCP
Access and utilization to EMR tools that help identify screening gap or indicate prior completed screening. Recent emphasis on increasing access to colonoscopy	“Systematically we are pretty good at reaching out to people and [we] have pretty good tools to identify them. We know who they are. We know what they need. And, we have a pretty good process to tell them what they need and to try to connect the dots for them.” —HP leader
Recent emphasis on increasing access to colonoscopy	
General surgeons and other staff trained in colonoscopies alleviated some resource/access constraints	“Fortunately, the backlog in GI is down quite a bit from what it used to be. When I first got here, it was a two year wait, and now it’s maybe three months. So it’s totally manageable since they have obtained enough manpower to actually do the testing, which is great.” —PCP
Organizational shift allowed more flexibility and support for referring patients’ to a screening colonoscopy, especially if patient requested	“Now I can refer them to colonoscopy. And with the FIT I can have these easier conversations. So I’m promoting FIT, but if they still want the colonoscopy, I’m going to refer for it.” —HP leader/PCP
Overall improvement in organizational CRC guidelines to make them more in line with national standards and emphasis on colonoscopy	“Until recently, the organizational recommendation was hemoccult testing and flex sig [sic]. And, that probably was not the community standard or the national standard… More recently, the GI Department has made colonoscopy more available. And I think that’s been a real advantage in my patient population and getting them screened.” —PCP

#### Preexisting integrated structure for dissemination of key practices

Health plan leaders cited their confidence in the abilities of key leaders, managers, and implementation groups within the integrated health system. PCPs emphasized the importance of experts in the system to facilitate CRC screening knowledge and programs. They also cited a strong trust in the skill level, training, and recommendations of endoscopy specialists in the organization. They also valued having a PCP champion within the system who could help colleagues both navigate and integrate new CRC research with organizational goals and patient preferences. Some PCPs also found that the EMR tools that alert them when CRC screening is due, or show indications of prior completed screenings, were facilitators in their screening efforts. Lastly, PCPs reported that use of support staff (medical assistants) trained in educating and motivating patients on screening and follow-up helped them to screen more patients.

#### Recent emphasis on increasing access to colonoscopy

Specialists identified the importance of increasing future access to colonoscopy and discussed solutions such as having general surgeons and other staff trained in conducting colonoscopies. Specialists described being motivated to support the program’s efforts because they see firsthand the consequences of colon cancer for patients. PCPs perceived a subtle organizational shift in recent years that had allowed them to more easily refer patients for screening colonoscopies, particularly if that was the patient’s preference. This, along with decreasing the wait time for screening colonoscopies, and adjusting the organization’s CRC guidelines to be more in line with the U.S. emphasis on colonoscopy, were also perceived by PCPs as important organizational changes that facilitated CRC screening.

### Lessons learned: implementation successes and challenges

#### Automated telephonic outreach successes and challenges

Health plan leaders felt the reminder calls had helped to improve the organization’s CRC screening rates, and said that calls are an effective, sustainable resource for supporting the organization’s efforts to screen a large population. They described the reminder calls as acceptable to patients, patient-centered, friendly, and easy to follow. PCPs said the reminder program helped to decrease workload for providers and health care teams by decreasing the need for individual providers to conduct their own outreach. Challenges to successful implementation of the centralized, automated reminder program identified by health plan leaders and managers centered on the interface of the program with the electronic medical record, with Medicare and Medicaid regulations, and with population health services sending kits to patients. They recommended involving the information technology (IT) department early in order to optimally integrate automated calls with the EMR. Health plan managers identified the challenge of being adequately staffed to quickly enter orders in the EMR and get them mailed to patients who wanted a stool test. This constraint initially decreased interest and compliance in completing or returning the stool test kits. They recommended streamlining the ordering of the stool test for patients due to receive a reminder call so the order would already be in the EMR, ready to be acted upon. They also suggested automatically sending stool test kits to every person who is due for screening following the receipt of a reminder call (Tables 3 and 4a, b).

**Table 3:** **Lessons learned: implementation successes (** ***n*** **= 55)**

**Summary of themes**	**PCP (** ***n*** **= 20)**	**Specialist (** ***n*** **= 23)**	**Leader (** ***n*** **= 12)**	**Sample of illustrative quotes (stakeholder group identified)**
Use of automated telephone outreach				
Helped to improve screening rates from previous years			✓	“I think the work that we’re doing in outreach with those modalities has been the reason we have significantly increased our screening rate… I think we’ve gone up six or seven percent in the last year.” —HP leader
An effective and resource sustainable method for increasing and maintaining CRC screening in a large population			✓	“I think it’s the way to address all kinds of things. And we’ve done it in a number of other areas… I think you want to have a centralized approach.” —HP leader
Patient-centered, friendly, easy to follow and use			✓	“It was amazing… we were able to keep members on the phone for up to five minutes because it was so interactive.” —HP leader
Decreased workload burden for providers/health care teams for conducting outreach calls for screening	✓		✓	“For colon cancer screening, what we pretty much have always done is in-reach during a visit… having an automated program makes it easier for us—especially for reaching those people whom we never see [in a visit] and tend to miss.” —PCP
Made PCP/health care team discussions with patients about CRC screening easier by reinforcing awareness and knowledge of importance of screening	✓		✓	“Ironically, lately I’ve been finding a lot of patients who, when I say, ‘Well, now we need to do that poop test.’ They’ll say, ‘Oh, I just turned that in.’ [Laughs] They’ve already done it… So it [a reminder program] just makes those conversations about screening easier.” —PCP
Use of fecal immunochemical tests				
Transition to FIT further increased the organization’s. CRC screening rate from prior years/increased patient compliance with the fecal test method of screening	✓	✓	✓	“And it’s just remarkable how many more of them are getting done. Now, part of that is that you only have to do one. You don’t have to do three. You don’t have to worry about the diet, like you did with the FOBT. So, it’s a lot easier, I think.” —HP leader
Adoption of FIT has given providers a fecal test method they have greater trust in and enthusiasm for due to increased patient compliance and test sensitivity	✓	✓	✓	“From a population perspective, it is the most effective because people will do it. And it’s easy, and it’s efficient. And it has literature to support it. So, it’s got all the right stuff.” —HP leader
Removed common barriers to fecal test completion for patients and made motivation/discussion about. CRC screening easier and more efficient	✓		✓	“But I think now, with the FIT test, it’s so much easier to have the conversation and just explain it’s different and really easy to use. How you collect your sample and how you send it out is so much easier. You don’t have to change your diet. So I think that has improved.” —HP leader
Communication about organizational screening approach				
Improved ability to provide a more unified message to all providers to encourage/discuss FIT for average-risk patients first, followed by offering colonoscopy if patient prefers or demands	✓	✓	✓	“It’s clear a lot of time and effort has been invested in communicating to Kaiser clinicians to see colonoscopy as not a better test than these other tools, and to offer stool card testing. I’ve probably been brought around to that line of thinking… I certainly think the newer stool card testing [FIT] has more merit… so it’s been a little bit easier for me to make my peace with that.” —PCP
Provides confidence in automated reminders, yearly FIT cards, and ongoing ability to offer screening colonoscopy	✓	✓	✓	“It’s a wonderful thing that we finally have turned on a screening program.” —GI specialist
Fewer organizational barriers to CRC screening than before implementation efforts	✓		✓	“Things are moving in a positive direction. I don’t see a whole lot of challenges necessarily, compared with a couple of years ago… I really think the barriers have been reduced. I think there’s been more provider satisfaction, and patient satisfaction as a result of those activities.” —PCP
Education and communication about resource stewardship and evidence based outcomes as it pertains to CRC screening seen as helpful	✓		✓	“Just recently, we’ve actually fed back to physicians, what their colonoscopy rate was versus their colleague who has the same risk adjusted population. And, some doctors were just mortified that they were sending out twenty times more than the doctor down the hall who had patients that weren’t that different… so as an organization, we owe all of our patients a research stewardship perspective.” *—*HP leader

Check symbol indicates theme brought up by more than half the stakeholder group.

**Table 4:** **Lessons learned: implementation challenges (** ***n*** **= 55)**

**Summary of themes**	**PCP (** ***n*** **= 20)**	**Specialist (** ***n*** **= 23)**	**Leader (** ***n*** **= 12)**	**Sample of illustrative quotes (stakeholder group identified)**
Use of automated telephone outreach				
Inadequate consideration of how the reminder program would interface with Medicare and Medicaid reimbursement regulations			✓	“I can’t overstate the importance of communication with External Affairs… in particular regarding Medicare guidelines about who is eligible for screening, and how reimbursement happened. So that was yet another whole layer of, okay, how do you [deal with] this so that the organization is in compliance with the federal regulation but isn’t burdening, you know, a thousand primary care clinicians. It… took a lot of work to get through that issue”. —HP manager
IT department not involved early enough in program development to determine how automatic calling system would interface with EMR			✓	“You need an analyst who can not just supply the data you ask for, but make sure that you’re asking for the right data, and [that the data] are really going to meet your needs… I think they need to be integrally involved in the planning process. As well, you need an implementation person… [who] can maintain a picture of what’s going on… because you don’t want the project to become siloized, with everybody just working independently”. —HP manager
Organization not prepared or staffed to meet the need for entering orders for patients who got fecal tests mailed after the reminder call			✓	“The analyst would put it into an Excel spreadsheet and then send a packet to our medical assistant, who would put in all those orders. Which could be, you know, eight hundred, twelve hundred orders. It’s a lot of ordering. However, recently, we have gotten a system in place that allows for batch ordering”. —HP manager
Slow response in mailing out fecal test to those patients saying “yes” during the call negatively affected patient compliance and interest once kits arrived at patient’s home			✓	“It would take sometimes up to six weeks to get these mailed out, because we couldn’t mail them out without an order, because the lab can’t do anything with a kit that comes back that doesn’t have an order”. —HP leader
Lack of integration or documentation of reminder calls in the EMR increased providers’ chance of not knowing a patient had been called	✓	✓	✓	The big deal [was] the complaints about not knowing which patients were called. And that’s just something that we can’t give them. But I think that that’s what leadership hears the most of”. —HP leader
Use of fecal immunochemical tests				
Need to improve clarity of instructions for fecal tests	✓			“I’ve had a number of patients tell me that the lab has said, don’t mail it back [fecal test]. You need to drop it back in. So I’m not sure if that’s an area that the organization has looked at… I’m not sure if our mailing package might need to change, or our instructions with the kit… But that would be one barrier to maybe getting it back if people have been told, either correctly or incorrectly, that they have to drop it off in person”. —PCP
No clear process for labeling kits, both when distributed centrally or when distributed from the point of care			✓	“We had some problems with FIT tests coming back unlabeled. I don’t think it was a lot, but it was enough”. —HP manager
System does not involve automatically sending fecal test kits in the mail to every person who is due following receipt of the automated reminder call			✓	“There are ways we can improve. I mean, we’re constantly kind of assessing… Southern Cal [Kaiser]… automatically sends the kit in the mail to every single person that’s due”. —HP manager
Communication about organizational screening approach				
Lack of effective and efficient ways to clearly communicate the organization’s CRC screening approach preferences to providers (PCPs/health teams/specialists)	✓		✓	“The challenge is always going to be making sure your physicians are excited about these kinds of screenings; not just for cancer, but for a variety of different things, and that they’re your best advocates… We need more of a unified voice behind our preferred screening modality”. —HP leader
Need for ways to effectively communicate and educate resource stewardship and evidence based outcomes to providers as they pertain to CRC screening		✓	✓	“If the patient wants a colonoscopy, that’s a very difficult discussion… because, if we’re still in the mode where we do what the patient wants, then we’re going to try to do [it] within a reasonable guideline. I don’t know how you remedy those two issues”. —GI specialist
Ongoing challenge of shifting the beliefs/habits of some providers (PCP and specialists) away from colonoscopy as the only appropriate screening choice for average-risk patients	✓	✓	✓	“I think it’s kind of a dilemma… If a friend of mine walks up and says, what test do you recommend to me? I would tell them colonoscopy… I think the colonoscopy is the best test”. —General surgeon
Need to clarify roles, processes, and expectations between PCP and specialist regarding CRC screening follow-up issues	✓			“One challenge that is sometimes unclear is who’s going to follow the referral [surveillance colonoscopy after a positive initial screening colonoscopy]. Do specialists automatically send follow-up to the patient that you need another one because this is positive [showed polyps], or are they expecting us [the PCP] to automatically re-refer them?” —PCP
Need for improvement in creating a service that integrates all components of the program, involving input and efforts of GI, surgery, oncology, and primary care		✓		“It’s an upgraded service program in the sense that you can’t do this without having oncology, surgery, GI and primary care [work] as an integrated team. I mean, the patient flow issue is related to both the screening program and the subsequent care. It’s not just one little cross sectional piece of care. It’s one piece of the integrated process”. —GI specialist
Concerns about screening duplication				
Patients new to the organization and with a recent negative colonoscopy being inappropriately given FIT kit		✓	✓	“We’ve seen any number of patients that come through with a positive FIT test who have actually had a negative colonoscopy within ten years. In my view… no one should be allowed to order another screening test [for them]”. —GI specialist
Lack of clarity on protocols and communication strategies by PCPs for patients with a negative FIT who also requested a screening colonoscopy		✓	✓	“A lot of people have been told by their primary care that if their FIT was negative they can’t get a colonoscopy. …They can. You just have to have it referred. There’s a several month waiting period. There are lots of messages sent to primary care about this”. —General surgeon
Approach of offering multiple screening methods and utilizing multiple outreach strategies of reminder calls and in-clinic prompting may be creating some screening duplication	✓	✓	✓	“Sometimes people get these stool cards at the Flu Clinic or by mail when they’ve already had a colonoscopy, or some other way they really shouldn’t have gotten one. And then they’ll bring them back and it’s positive”. —PCP
Need to standardize documentation in EMR of patients’ prior CRC screening and related result so there is clear and easy access to information for all providers	✓	✓		“Sometimes it’s difficult using [EMR] what type of screening has previously been done. I’ve had referrals sent to me where someone gets referred for a colonoscopy and they had one three years ago… So far we don’t have a system-wide way to write it in the problem list. We’re trying to standardize that. And finding the notes when you’re just scanning the charts is very, very difficult… even if a physician is trying to really find that, it’s hard”. *—*GI specialist
Ongoing need for education				
More patient education about CRC screening that can be delivered by support staff (MAs and RNs)	✓		✓	“Some patient education materials would be nice… anything that would summarize the pros and the cons of the different types of screening. And it wouldn’t be a bad idea for some of that material to be handed to the patients by support staff, so that while they’re waiting in the room they could look it over and then maybe be a little bit informed before the office visit”. —PCP
Create more consistent, uniform, centralized messages utilizing a variety of methods (e.g., visual aids for patient navigation, provider decision-trees, etc.)	✓		✓	“What might be helpful is if I had a FAQ sheet [for PCP] like what is the incidence of colon cancer for average risk patients, fifty to sixty, sixty to seventy, etc. What is the risk if there is a family history? And possibly a fact sheet for patients too, because it is definitely the patients who leave here who are undecided and they struggle or they have questions”. —PCP
Direct patients with a recent normal colonoscopy not to get a fecal test (FIT)		✓		“There [needs] to be a big bullet on the FIT test that says, if you had a normal colonoscopy within the last five years, throw this away immediately. These are automatically mailed out to patients who the year previously had a normal colonoscopy. Five to eight percent are positive, then they’re wanting another colonoscopy”. —General surgeon
Proactively educate patients about choices and controversies related to screening		✓		“Anything that can be done to provide the patient with information about the controversy or choices, or how to pick up or get a test done… You take your FIT test and it is positive, this is what will subsequently [occur] in your care. So the patients sort of know where they’re going to go with this, what the expectations are, and what Kaiser will provide to them”. —General surgeon
Increase staff and colonoscopy resources/access				
Increased sensitivity and compliance of FIT. unintentionally created resource and access issues again with colonoscopy		✓	✓	“Because we’re screening more people, we’re finding more positive FITs and it’s driving our colonoscopy rates up. But they’re driven up appropriately… but this will require us to, again, strategize about what we’re going to do as an organization”. —HP leader
Continuing need for additional highly trained staff (including mid-level providers) to do screening colonoscopies, helping to improve wait-times and access	✓		✓	“We’re going to need more people capable of doing a good colonoscopy. We’ve been at the forefront in the past of hiring PAs and training them to do that. And right now there some considerations to do it, but that’s a big political thing”. —HP leader
Need to make CRC screening a self-referral program, similar to other screening programs (e.g., breast cancer screening)		✓		“Make it self-referral”. —General surgeon

Check symbol indicates theme brought up by more than half the stakeholder group.

#### Fecal immunochemical tests

Health plan managers felt that implementation of FIT increased patient compliance with the stool test method of screening; instructions for completing FIT were perceived as being easier for patients to follow. Yet, both health plan leaders and PCPs felt there was room for improving the readability of instructions and protocols for distribution of kits; for example, some kits distributed in clinic were returning to the lab without proper patient identification labels. Health plan leaders felt that the transition to FIT further increased the organization’s CRC screening rate from prior years. PCPs and health plan managers described how ease of FIT completion made discussion about CRC screening easier and more efficient. Additionally, PCPs said that increased test sensitivity of FIT has raised their enthusiasm for, and trust in, stool test screening. Specialists expressed enthusiasm about having a centralized screening program, but also ambivalence about prioritizing fecal testing over colonoscopy.

#### Communication about organizational screening approach

All three stakeholder groups felt the organization had improved its communication so to provide a unified message to providers to encourage and discuss FIT screening as an option, and offer screening colonoscopy if the patient preferred this option. Nonetheless, all stakeholder groups felt there were still some providers who believed that colonoscopy is the only acceptable method for CRC screening for average-risk patients. Health plan leaders and PCPs both felt the organization continues to lack effective and clear methods to communicate the organization’s CRC screening approach to all providers. Specialists said there was a need for communication to help staff see the screening program as an integrated service involving input and effort from multiple departments such as surgery, gastroenterology, primary care, and oncology. PCPs and health plan leaders identified the helpfulness of education on resource stewardship and on feedback of providers’ screening tendencies. Yet, health plan leaders expressed the desire for more effective ways to communicate and educate providers about resource stewardship issues and evidence-based outcomes as they pertain to CRC screening.

#### Concern about screening duplication

There was concern from all three stakeholder groups that the current CRC program may be creating some screening duplication, contributing to inefficient use of resources. Health plan leaders endorsed the multimodal approach to offering screening options to patients (i.e., at patient visits (in-reach) and via automated calls (outreach) as important attributes of improved screening rates. Yet, health plan leaders and PCPs identified that the lack of integration or documentation of the reminder calls in the EMR created challenges by increasing providers’ chances of duplication of effort or service. PCPs desired clearer prompting through the EMR about patient screening status (in particular, whether they had received an automated call) during the clinic visit. Specialists felt strongly that improving EMR alerts and directions for providers/support staff could also prevent patients who had a recent normal colonoscopy from receiving a FIT. PCPs and specialists found overall documentation of prior colonoscopy results to be inconsistent and confusing, as they could not always discern what were recommended next steps for future screening. Specialists were frustrated that some patients were still inappropriately screened, such as the very elderly or hospice patients. Concern about screening duplication generated questions where evidence-based practices and policies were unclear. For example, specialists and health plan leaders described a lack of clarity on protocols for patients with a negative FIT who requested a screening colonoscopy within the same year.

#### Ongoing need for education

PCPs and health plan leaders both underlined the need to improve patient education regarding CRC screening importance and options. PCPs requested more consistent educational tools to help patients navigate screening information and options (e.g., visual aids for discussion, exam-room posters, and patient handouts). Specialists emphasized the need to proactively educate patients about all available CRC screening choices. PCPs and health plan leaders agreed on the need to increase and standardize training of support staff in order to expand staff roles in CRC screening, since support staff are often the ones who see patients first and place the screening order in the system; they suggested enabling preordering of the screening test during rooming procedures.

#### The need to increase staff and colonoscopy resources and access

The greatest challenge to implementing FIT, as identified by health plan leaders and specialists, was unintentionally creating resource and access issues with colonoscopy services due to the increased compliance with, and sensitivity of, the test. Health plan leaders and PCPs felt that there is still a need for additional highly trained staff to provide endoscopy services, including colonoscopies. PCPs advocated for the organization to train more mid-level providers to conduct colonoscopies to assist with issues of access. Specialists emphasized their desire for patients to be able to self-refer for CRC screening, similar to the breast cancer screening program already in place. Self-referral would allow the patient to choose any of the tests available and have direct access to scheduling, rather than requesting a referral from a primary care provider. At the time of this publication, the health system does not offer self-referral for endoscopy.

## Discussion

Prior studies have evaluated implementation of colorectal cancer screening programs within medically underserved populations in the U.S. [35-37], whereas this study specifically focuses on the effects of an innovative quality-improvement program to improve screening rates among members of an integrated health plan that serves mostly insured individuals. The multimodal quality-improvement approach was aligned with the PRISM framework, [22] which emphasizes implementation of multiple changes at once (or serially) while specifically taking into account the strengths and needs of the delivery system; Table 5 summarizes the facilitators and barriers to implementation of the program, for each of the PRISM domains. A key aspect of the program was a centralized automated telephone calling campaign, which we demonstrated previously to independently increase screening rates about 6.5% [19]. During the 2 years of implementation of the entire quality-improvement program, from 2008 to 2010, colorectal cancer screening rates increased from 54.8% to 65.9%. They have continued to rise, and in 2013 reached 80% or above.

**Table 5:** **Reported facilitators and barriers to program implementation, by PRISM domains**

**Core PRISM domains**	**Facilitators**	**Barriers**
Program (intervention) domain	• Centralized screening outreach addressed primary care time constraints in offering screening	• Optimal choice of screening test (i.e., fecal testing or endoscopy) was unclear from evidence
	• Adoption of FIT gave providers a fecal test method that they could more easily explain, addressing primary care time constraints	• Information technology department was not involved early enough in the process to determine best interfaces with EMR
	• Improved accuracy of FIT enabled communication of more unified message about screening prioritization within the organization	• Slow response in mailing out fecal tests to those that accepted outreach impacted the efficiency of the program
	• Incorporating automated screening reminder alert into electronic medical record built upon existing “care gap” reminder structure	• Increased compliance with new FIT kit unintentionally created access challenges with colonoscopy services for a while
	• Incorporating automated screening reminder alert enabled support staff to offer screening during primary care office visits	
External environment domain	• There was interest in increasing quality performance numbers (e.g., HEDIS measures) to the levels of those of other comparable health care organizations	• Alignment of automated reminders and fecal test orders with Medicaid and Medicare reimbursement regulations was challenging
Implementation infrastructure and sustainability domain	• Dedicated team for implementation had prior experience in implementing automated reminder programs for other health screening services	• There was a need to improve integration of program (e.g., documentation of centrally mailed FIT) within EMR
	• Data showing increased screening rates supported effectiveness of program	• There was a need to improve staffing levels and training for ordering/mailing FIT kits centrally, and tracking diagnostic follow ups
	• Recent emphasis on increasing capacity for colonoscopy enabled program to absorb increased number of colonoscopies	• There was a need to improve workflows and EMR documentation to decrease screening duplication errors
	• Cross-department support and coordination between population care leaders, information technology, laboratory services, GI department, PCPs and support staff enabled maintenance and improvement of program	• There was a need to improve FIT kit instructions and labeling of FIT kits to decrease errors in test completion and processing
Recipients domain	• Strong leader, manager, clinician, specialist and frontline staff belief in the importance of CRC screening facilitated program acceptance	• There was an ongoing need to continue education and to shift habits of some providers/specialists away from colonoscopy as the only screening choice
	• An historical cultural emphasis on screening helped the intervention to be perceived as an effective and important strategy worthy of continuing	• There was an ongoing need to clarify roles, processes and expectations between providers and specialists regarding positive screening follow-up issues
	• Providers and staff felt more trained on and educated about CRC screening options and resource stewardship issues	• There was a continued need to provide performance data feedback and clear expectations regarding CRC screening rates and organizational preferences to all staff

Unique to this study setting was a preexisting sophisticated electronic medical record (EMR), which integrates data from all departments and all sites of care. Stakeholders attributed screening increases in part to implementation of clinical decision support in the EMR, which allowed notification in the chart of patients who appeared overdue for screening, and enabled distribution of FIT kits from different points of clinical care. While outreach efforts from clinic support staff (through reminder letters) were already underway prior to the implementation, the addition of the centralized automated telephone outreach program enabled multimodal communication with, and reminding of, patients, which has been shown to enhance screening rates in prior studies [38-44]. Yet, participants also reported a continued need to refine the integration of the program with the system, both for outreach and for ensuring completion of stool tests.

Participants stated that changing from guaiac fecal occult blood test cards to the FIT test, a single-sample fecal immunochemical test with implementation of a centralized outreach program, provided much-needed clarity about organizational priorities. Prior to implementation, administrators expressed that it was difficult to know which screening test to prioritize for promotion across the system. U.S. guidelines do not strongly recommend one test over another [5,6]. Even with improved screening rates, stakeholders in different departments expressed continued ambivalence and disagreement about how to communicate about the different options available to patients for colorectal cancer screening (e.g., fecal tests and endoscopy). Some specialists and PCPs wanted to make screening colonoscopy more readily available to interested patients (e.g., through self-referral). Communication with program champions in the system helped resolve some of these disagreements, and has been described as a key facilitator of CRC program implementation [35,45]. Ongoing discussions about the changing landscape of CRC screening evidence and best practices will likely continue to be important in the coming years with the completion of large trials comparing FIT to colonoscopy [46,47].

An unintended consequence of program implementation was more screening duplication. Stakeholders reported that the ease of access and ubiquitous distribution of FIT led to some waste of fecal test kits and also to some over-screening. This finding is a downside to the benefit of achieving high screening rates by using multimodal reminders. PCPs and health plan managers reported that some patients received several kits at different points of care, because of electronic alerts indicating that he/she was overdue for screening. Those with low screening adherence would sometimes lose kits or would keep more than one at home, incomplete. Prior studies have targeted non-adherent patients by employing tailored navigation through telephone outreach by a nurse or medical assistant, though success of this approach has been variable, and widespread implementation would need to consider cost-benefit tradeoffs [48,49]. Screening overuse stemmed from patients who were willing to screen whenever prompted, sometimes completing more than one kit per year, or completing a colonoscopy less than 10 years after a prior negative colonoscopy. Stakeholders reported that incomplete documentation in the EMR of prior colonoscopy outside the health plan contributed to over-screening of this nature, which has been a previously reported challenge in other systems [50]. In theory, additional clinical alerts [45,50-54] at the point of ordering a fecal test kit would address both the duplication in fecal test kits and inadequate documentation of prior colonoscopy. However, computer-alert “fatigue” should be a consideration in implementing additional electronic prompting and oversight. Previous research has shown that complex clinical decision support initiatives to support CRC screening can result in a subsequent decline in quality outcomes due to this phenomenon [55].

Lastly, in spite of the focus of the program being fecal tests, stakeholders at all levels discussed the ongoing need to expand colonoscopy resources, namely, increasing the number of endoscopists and the dedicated facilities and staff for offering this procedure. Both administrators and specialists had concerns about limitations in colonoscopy resources, and how the organization should manage these resources while still offering screening to all eligible patients. Resource constraints are likely to remain an ongoing concern in colorectal cancer screening programs [56,57].

### Limitations and strengths

Our study could not discern whether stakeholders’ responses represent the feelings of non-interviewed clinicians, specialists, and health plan managers across the Kaiser Permanente system. However, responses were sufficiently consistent that we could identify themes and patterns across different groups. Another limitation may be that the lessons learned in this system, which has internal practice guidelines and a sophisticated electronic medical record, may be difficult to apply in systems lacking those attributes. However, ~80 million people in the U.S. receive care from an HMO, which likely have similar organizational attributes as those described here. Two strengths of this qualitative study are that we obtained a variety of viewpoints through multiple interviews, and that we elicited responses at a variety of time points relative to the quality interventions. We also used a prespecified interview guide, a trained interviewer, and a repeated, iterative process of reliability-checking among members of the research team.

## Conclusions

Our analyses of multiple stakeholder viewpoints about quality-improvement efforts to raise CRC screening rates yielded valuable information about successes, and remaining challenges, to successful implementation of a reminder program in a highly integrated U.S. managed health care system. CRC screening rates improved 10% during program implementation and continue to rise. The implementation program capitalized on historical facilitators (an integrated health system with internal guidelines and a sophisticated electronic medical record), while also removing some historical barriers to screening (organizational resource, staff, and time burden; patient non-compliance). Success was due in large part to the activation of three different domains within the PRISM framework: changing the delivery system design through centralizing screening efforts (implementation infrastructure); switching to a more accurate and feasible fecal test (external environment); and providing educational and electronic support (recipients of intervention). The combination of these actions resulted in a successful and sustained improvement in CRC screening rates.

Health plan leaders, PCPs, specialists, and support staff in other health systems can use our results to address remaining barriers to raising CRC screening rates. All the stakeholders we interviewed acknowledged the complexity of understanding and applying the scientific evidence for CRC screening, and expressed a need for ongoing education of both providers and patients about organizational emphases on CRC screening. They also wanted to understand, and have input into, the scientific reasoning informing an organization’s choices in recommending one test over others for people at average risk of colon cancer. Further refinement of the stool test distribution system should focus on reducing duplication of test dissemination, and installing new procedures for identifying and documenting prior endoscopies done outside of the system. Perhaps the greatest challenge to the system we studied is that increases in FIT testing, and the greater sensitivity of FIT, have led to greater demand for colonoscopies. Ultimately, decision-makers will need to re-examine the relative benefits of joint informed decision-making (specifically regarding patient choice of screening test) and weigh them against the benefits of centralized screening efforts through a single test (e.g., fecal immunochemical testing), and concentrate their resources accordingly.

## Abbreviations

CRC: Colorectal cancer
PCP: Primary care physician
FOBT: Fecal occult blood testing
FIT: Fecal immunochemical test
HMO: Health maintenance organization
KPNW: Kaiser Permanente Northwest
EMR: Electronic medical record
PRISM: Practical robust implementation and sustainability model
